# Management of Pigmented Gingiva in Child Patient: A New Era to the Pediatric Dentistry

**DOI:** 10.5005/jp-journals-10005-1218

**Published:** 2013-10-14

**Authors:** Rakesh Namdeoraoji Bahadure, Parul Singh, Eesha Jain, Heena Khurana, Gautam Badole

**Affiliations:** Lecturer, Department of Pedodontics and Preventive Dentistry, Sharad Pawar Dental College, Wardha, Maharashtra, India, e-mail: mdsrakesh_pedo@yahoo.co.in; Lecturer, Department of Pedodontics and Preventive Dentistry, FODS Chhatrapati Shahuji Maharaj Medical University, Lucknow, Uttar Pradesh, India; Postgraduate Student, Department of Pedodontics and Preventive Dentistry, FODS, Chhatrapati Shahuji Maharaj Medical University, Lucknow, Uttar Pradesh, India; Postgraduate Student, Department of Pedodontics and Preventive Dentistry, FODS, Chhatrapati Shahuji Maharaj Medical University, Lucknow, Uttar Pradesh, India; Lecturer, Department of Conservative Dentistry, VSPM Dental College, Nagpur, Maharashtra, India

**Keywords:** Depigmentation, Melanin, Gingiva, Pigmentation

## Abstract

Gingival health in the form of size, shape, consistency and appearance are essential components responsible for an attractive smile as well as may cause unpleasant appearance. Melanin pigmentation often occurs in the gingiva as a result of an abnormal deposition of melanin which can compromise the confidence level from the age of childhood. The present article describes and discusses the two cases of gingival melanin pigmentation in 12 and 13 years of female patient and their early surgical intervention with successful follow-up of 9 and 6 months. Patients were instructed to prevent sun exposure, intake of hot foods or beverages like cold drinks, tea, coffee and brushing immediately after surgery.

**How to cite this article:** Bahadure RN, Singh P, Jain E, Khurana H, Badole G. Management of Pigmented Gingiva in Child Patient: A New Era to the Pediatric Dentistry. Int J Clin Pediatr Dent 2013;6(3):197-200.

## INTRODUCTION

Gingival pigmentation occurs as diffuse purplish discoloration or as irregularly shaped brown or light brown patches. This pigmentation may be seen across all the races,^1^at any age^2^ and it is without gender predilection.^3^ It is more prominent in the black individuals with uneven distribution of the pigmentation in oral cavity occurs as gingiva 60%, hard palate 61%, mucous membrane 22% and tongue 15%. ^4^For depigmentation of gingiva different treatment modalities have been reported like bur abrasion, scraping, partial thickness flap, cryotherapy, electrosurgery and laser but repigmentation of the melanin pigment after surgical procedure is noted and considered in a high percentage.^5^Different factors as chemical, surgical technique, thermal and idiopathathic factors are responsible for recurrence.^5^So special precautions should be taken to delay the chances of recurrence after surgical intervention of pigmented gingiva.

## CASE REPORTS

### Case 1

A female patient 12 years of age reported to the department of pedodontics and preventive dentistry with the complaints of melanin pigmentation on gingiva. On examination melanin deposits was seen all over the gingiva with gummy smile exposing the gingiva from incisor to molar region. Shyness and low confidence level of patient was observed while examination.

**Fig. 1:**
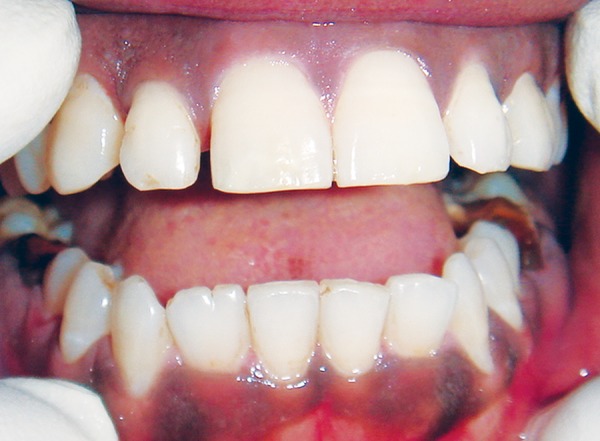
Preoperative view of pigmented gingiva

**Fig. 2:**
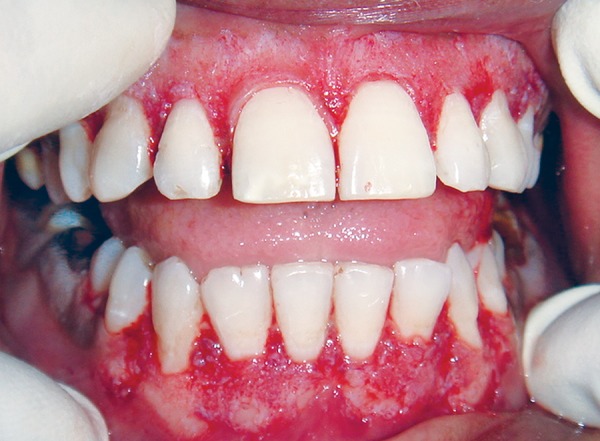
Gingiva scaping during operative procedure

**Fig. 3:**
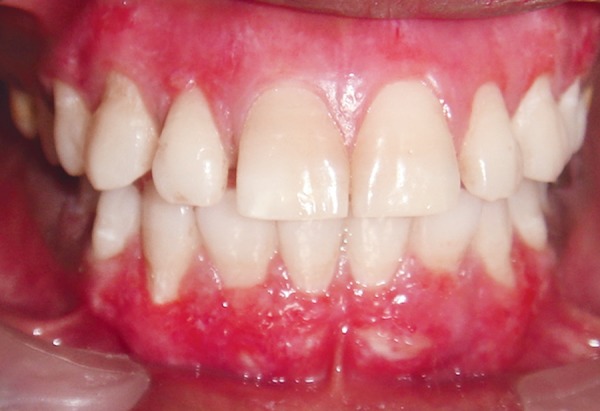
Follow-up after 7 days of scraping procedure

**Fig. 4:**
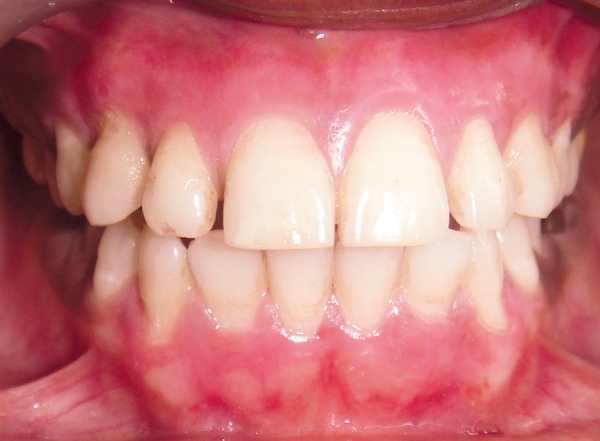
Follow-up after 9 months of scraping procedure

The patient consent was taken. After oral prophylaxis and maintenance of oral hygiene instructions the surgical procedure was carried out under local anesthetic infiltration. The pigmented gingival epithelium from distal of first premolar from one side to distal of first premolar of the opposite side was excised by scraping of epithelium, using number 15 and 12 BP blades in both the arches. It was carried out in scalloping pattern. Any remnants of the pigmented areas that were left were removed. The follow-up of 9 months showed no pigmentation reappearance (Figs 1 to 4).

### Case 2

**Fig. 5:**
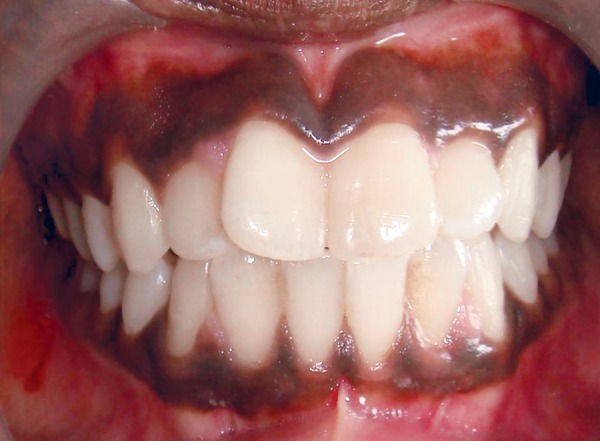
Preoperative view of pigmented gingiva

**Fig. 6:**
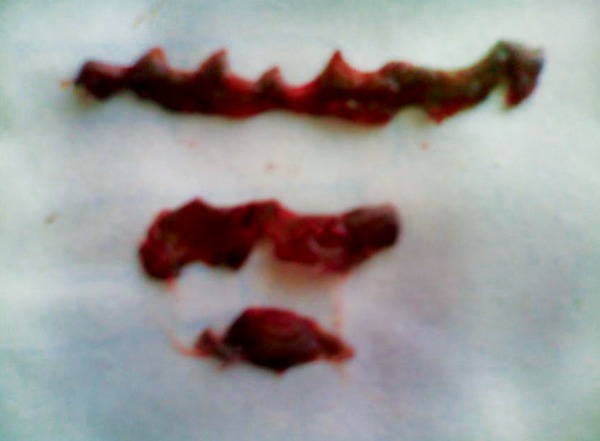
Partial thickness straps of pigmented gingiva

**Fig. 7:**
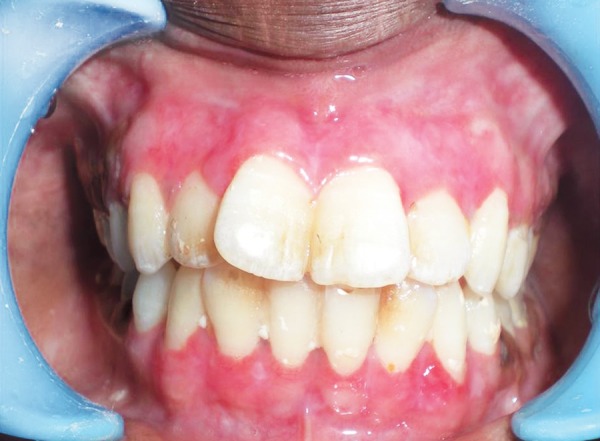
Postoperative photograph

A 13-year-old female patient visited to the department of pedodontics and preventive dentistry with the complaints of melanin deposits on the gingiva. In this case we were planned to remove the partial thickness flap of pigmented gingiva. The patient consent was taken. After oral prophylaxis and maintenance of oral hygiene instructions the procedure was done under local anesthetic infiltration. The pigmented gingival epithelium from distal of first premolar on one side to distal of first premolar of the opposite side was excised by removal of complete epithelium and some part of connective tissue strap using number 15 and 12 BP blades in both the arches. Postoperative normal healing was observed (Figs 5 to 7). Follow-up of 6 months showed the normal pinkish appearance of gingiva.

### DISCUSSION

Gingival pigmentation in the form of melanin deposits generally observe in all the normal individuals but most of time it is not detectable clinically. Physiological pigmentation in the form of melanin is nonhemoglobin derived brown pigment and is responsible for normal pigmentation of the skin, oral mucosa and gingival tissue. But it is considered to be multifactorial, whether physiological or pathological and can be caused by a variety of local or systemic factors. Durnmet 1979 included genetic, tobacco use, prolonged administration of certain drugs especially antimalarial agents and tricyclic antidepressants^6^ in his study as etiological factors. In the present cases we did not observed any history of environmental causes. In family it was observed in mother but not in such severe form. In second case the family history was not relevant.

There are different techniques to remove the gingival pigmentation as through surgery^7^ lasers^3^, cryosurgery through use of a gas expansion system,^8^ bur abrasion, scrapping and electrocautery have been reported by Hirschfeld and Hirschfeld 1951 and many other authors. Hirschfeld and Hirschfeld (1951) used phenol (90%) and alcohol (95%) to remove areas of oral pigmentation by destroying tissue down to and slightly below the basal layer of the mucous membranes. Repigmentation soon developed in three patients; the rest of the subjects met with the same results a short while later. Durnmett and Bolden (1963) operated pigmented gingiva by gingivectomy procedure in 9 cases. Repigmentation occurred in 67% of the areas, as early as 33 days after surgical removal. A study by Oswaldo et al, 1993 showed that gingival surgical procedures performed solely for cosmetic reasons, offer no permanent results. Spontaneous repigmentation has been shown to occur and the mechanism suggested is that the melanocytes from the normal skin proliferate and migrate into the depigmented areas. Most of the authors concluded that pigmented areas are present only when melanin granules synthesized by melanocytes are transferred to the keratinocytes. This close relationship between melanocytes and keratinocytes was labeled by Fitzpatrick and Breathnach (1963) as the epidermal-melanin unit.^9^ Further research is required on repigmentation to study the factors affecting rate and length of time required for recurrence of pigmentation. In the present cases we used the surgical procedure with minimal trauma in both techniques and postoperative instructions as to avoid using hot foods, any chemicals, prevent sun exposure as much as possible or use of umbrella, avoid tea or coffee any cold drinks for some time after surgery. It will somewhat help to reduce the reappearance of melanin pigments or increase the duration of success.

In one study it was observed that electrosurgery showed good results, followed by epithelial excision and bur abrasion-scrapping techniques. The sites operated with bur abrasion-scrapping method showed a slight recurrence of pigments than the other two methods. The pattern observed was patchy in distribution and had occurred by 3 months.^10^Another effective treatment for depigmentation is using lasers. A one step laser treatment is available usually sufficient to eliminate the pigmented areas and do not require any periodontal dressing. This has the advantages of easy handling, short treatment time, hemostasis, decontamination and sterilization effects. In the present cases we found the 9 month and 6 month successful follow-up. But still long term follows-up and advance research is required in this field for permanent solution.

As concluded by most of author that early age intervention of gingiva may reduce the thickness of attached gingiva. In the present cases we tried for minimum trauma with only epithelium in one case and epithelium with slight layer of connective tissue in partial thickness flap were removed in another case. Early intervention is carried to find out the success rate of early intervention in the management of pigmented gingiva, to improve the esthetic appearance for long duration.

## CONCLUSION

The techniques with minimum trauma, surgical intervention in young age and postoperative precautions may improve the result and delay the reappearance.
